# Placenta Prævia Complicated with Hydatiform Cysts

**Published:** 1886-06

**Authors:** R. H. L. Bibbs

**Affiliations:** Saltillo, Mexico


### A CASE OF PLACENTA PRÆVIA, ASSOCIATED WITH HYDATIFORM TUMORS, ETC.

*Reported by R. H. L. Bibbs, M. D. Saltillo Mexico.*

(For Daniel's Texas Medical Journal.)

ON the 25th of last May, I was requested to take charge of Senora C. a primipara, aged 23 years, who menstruated last, on the 1st of November preceding, and was married on the 20th of the same month. With exception of nausea and vomiting, patient had noticed no deviation from her usual health until the latter part of March, from which date, she had been continually "loosing blood." From about the middle of April, she had suffered from irregular chills, sweatings and a fetid, sanious discharge, frequently associated with large quantities of biood. Family history of herself and husband, good—negative as regards seyphilis.

Her condition at the date of my first visit, was decidedly septicæmic; eye-lids, face and feet œdematous; abdomen, large and tender; frequent vomiting and diarrhœa; tongue, pale and flabby; pulse, 140 and gaseous; temperature, 103; countenance, anxious and waxy; she was flowing considerably and every movement provoked attacks of syncope.

From a digital examination, I found the placenta firmly attached to, and around the internal os—the back of it occupying the right side of the uterus; on the left side, it was detached, and my finger readily entered the cavity of the organ. The uterus and the vagina were hot and extremely tender, and the parts were bathed in an offensive ichorous discharge. Auscultation failed to detect the foetal heart, and the placental souffle was heard very indistinctly.

I explained to the family—a very intelligent one—that the patient was in a dangerous condition; that she had placenta prævia and was suffering from septicæmia caused by the retention of putrescent products of conception, and that in my opinion the only hope—even if the case admitted any reasonable grounds of hope—consisted in speedy evacuation of the uterine contents, and requested that Dr. Smith be called to my assistance.

Pending arrival of Dr. Smith, and in obedience to the indications suggested by her condition, as well as to fortify the patient for another examination, I gave her, hypodermically, a sixth of a grain of morphine and a hundreth of a grain of atropine and ordered iced champagne freely. Under this,. the patient revived, so that, by the time the consulting physician arrived, she expressed herself as feeling much stronger and entirely free from the previous disposition to syncope.

Dr. Smith's examination confirmed the diagnosis already expressed, and concurring with me in treatment, we determined to repeat the injection of morphine and atropine and to dilate and empty thc uterus at once.

Acting upon this determination, the patient being brought under the influence of the anæsthetic mixture used at Grey's Hospital, London—alcohol, ℥i chloroform, ℥ii sulph aether ℥iii—I introduced my hand, previously annointed with cosmoline, gradually dilated the uterus, broke up the placental attachments and extracted, in conjunction with a gallon and a half, or more, of hydatiform cysts, a large, well formed decomposing female fœtus, apparently in the fifth month of gestation.

My hand was again introduced into the uterus, carrying with it, the tubing of a fountain syringe, by means of which, the organ was thoroughly washed out with *hot water* containing bichloride of mercury in the p oportion of one part per thousand. There was considerable hemorrhage, but under the stimulus of the *hot water* and ergotine hypodermically, the uterus contracted well—expelling my hand, which brought away all debris, thus leaving the organ in the most favorable condition possible, under the circumstances.

The patient was cleansed, wrapped in warm blankets and put to bed. Reaction, which was slow, was produced by friction with dry mustard, application of bottles of hot water, and hypodermic injections of ammonia, brandy and caffein—she being unable to retain anything taken by the mouth.

Twice during the operation, which was prosecuted with as much haste as was deemed compatible with safety and thoroughness, the extreme debility of pulse—from which, I thought, there could be no reaction—necessitated the exhibition of ammonia by inhalation, and of ammonia and brandy by the hypodermic method.

The subsequent progress of the case, under favorable hygiene, daily uterine irrigations with the bichloride of mercury, the free use of stimulants, chiefly champagne, administration of iron and quinine, with an occasional injection of morphine, although tedious and slow, was to perfect recovery, and the patient was discharged restored, on the 15th of July following.

Quite a number of the cysts were subjected to microscopical inspection, but the total absence of hooklets and other evidences of the cysticurcus, left no room to doubt their hydatiform identity.

It may not be out of place, since many of my Texas confreres are occupying themselves somewhat extensively in studying the application of electricity to conditions often met with during pregnancy and parturition, to state that, when this patient began to "flood" in March, she was treated by a Mexican physician who informed her she had aborted, and that he would apply electricity to produce uterine contractions. The family noted that, while there had been much hemorrhage, clots of bloods, etc., there had been no appreciable diminution in the size of the patients abdomen, and requested the physician to explain the phenomenon, which he did, by saying the case was one of superfœtation in which one embryo had been expelled, and the other had remained, and would go on to full term if he was permitted to continue the daily use of the electric cure, which was consented to, and faithfully applied, up to the date of my first visit—with the result, as the family believe, of producing the death of the remaining embryo, and causing the growth of the hydatids removed by me on the 25th of May.

